# Infant vaccination timing: Beyond traditional coverage metrics for maximizing impact of vaccine programs, an example from southern Nepal

**DOI:** 10.1016/j.vaccine.2015.12.061

**Published:** 2016-02-10

**Authors:** Michelle M. Hughes, Joanne Katz, Janet A. Englund, Subarna K. Khatry, Laxman Shrestha, Steven C. LeClerq, Mark Steinhoff, James M. Tielsch

**Affiliations:** a Johns Hopkins Bloomberg School of Public Health, Department of International Health, Global Disease Epidemiology and Control, 615 North Wolfe Street, Baltimore, MD 21205, USA; b University of Washington, Seattle Children's Hospital, 4800 Sand Point Way NE, MA 7.234, Seattle, WA 98105, USA; c Nepal Nutrition Intervention Project – Sarlahi, Kathmandu, Nepal; d Tribhuvan University Teaching Hospital, Department of Paediatrics, Institute of Medicine, Maharajgunj, Kathmandu, Nepal; e Cincinnati Children's Hospital and Medical Center, Global Health Center, R8508, 3333 Burnet Avenue, Cincinnati, OH 45229, USA; f George Washington University Milken Institute School of Public Health, Department of Global Health, 950 New Hampshire Avenue, Washington, DC 20052, USA

**Keywords:** Childhood immunization, Vaccination timeliness, Vaccination coverage, Nepal

## Abstract

**Background:**

Immunization programs currently measure coverage by assessing the proportion of children 12–24 months who have been immunized but this does not address the important question of when the scheduled vaccines were administered. Data capturing the timing of vaccination in first 6 months, when severe disease is most likely to occur, are limited.

**Objective:**

To estimate the time to Bacillus Calmette–Guérin (BCG) (recommended at birth), diphtheria-tetanus-pertussis-H, influenza b-hepatitis B (DTP-Hib-HepB), and oral polio vaccine (OPV) (recommended at 6, 10, and 14 weeks) vaccinations and risk factors for vaccination delay in infants <6 months of age in a district in southern Nepal where traditional coverage metrics are high.

**Design/methods:**

Infants enrolled in a randomized controlled trial of maternal influenza vaccination were visited weekly at home from birth through age 6 months to ascertain if any vaccinations had been given in the prior week. Infant, maternal, and household characteristics were recorded. BCG, DTP-Hib-HepB, and OPV vaccination coverage at 4 and 6 months was estimated. Time to vaccination was estimated through Kaplan–Meier curves; Cox-proportional hazards models were used to examine risk factors for delay for the first vaccine.

**Results:**

The median age of BCG, first OPV and DTP-Hib-HepB receipt was 22, 21, and 18 weeks, respectively. Almost half of infants received no BCG by age 6 months. Only 8% and 7% of infants had received three doses of OPV and DTP-Hib-HepB, respectively, by age 6 months.

**Conclusion:**

A significant delay in receipt of infant vaccines was found in a prospective, population-based, cohort in southern Nepal despite traditional coverage metrics being high. Immunization programs should consider measuring time to receipt relative to the official schedule in order to maximize benefits for disease control and child health.

## Highlights

• We prospectively examined, via weekly recall, the timing of EPI immunizations in infants less than 6 months in rural Nepal.
• The majority of infants less than 6 months received immunizations on a delayed schedule.
• National immunization coverage estimates do not capture delay in the first 6 months of life.

## 1 Introduction

Immunization is the primary means of prevention for several childhood infectious diseases. Approximately 2–3 million deaths are prevented each year due to immunization with diphtheria, tetanus, pertussis, and measles vaccines [1]. Since the introduction of the Expanded Programme on Immunization (EPI) in 1974 the percentage of children protected against six diseases (tuberculosis, diphtheria, tetanus, pertussis, polio, and measles) increased from 5% to 83% (measured at 12–23 months of age) [2], [3], [4]. For example, the World Health Organization (WHO) estimates that since the end of the 1980s, 80% of children worldwide received pertussis vaccines, preventing approximately 38 million cases and 600,000 deaths annually [5]. Despite tremendous progress, global coverage remains below the target of 90% diphtheria-tetanus-pertussis-3 (DTP3) coverage [6]. While EPI has dramatically reduced the incidence of vaccine-preventable diseases they remain an important contributor to child deaths in low and middle-income countries [7].

Delay in vaccination is especially important for infants who are generally at high risk for severe morbidity and mortality from these diseases [8]. While infants might have partial protection from passive transfer of antibodies from their mothers, this immunity eventually wanes, requiring active immunization for infants to be protected against disease [9].

In Nepal, DTP3 vaccine coverage increased from 54% of children fully vaccinated by 12–23 months of age in 1995 to 90% in 2012; similar increases were seen for oral polio vaccine-3 (OPV3) (50–90%) and Bacillus Calmette–Guérin (BCG) (76–96%) [10]. Even though current coverage is high, this measure does not capture the timing of vaccine receipt relative to the official schedule. Recent estimates of coverage at 6 months in low and middle-income countries found DTP3 coverage was just 36% and BCG coverage was 85% [11]. A focus on vaccine receipt as close as possible to the official schedule could significantly improve the benefits of immunization programs. Unfortunately, population-based data on early vaccination coverage using active surveillance in low-income countries are lacking. This prospective, population-based cohort study aimed to estimate vaccination timing and risk factors for delay in the first 6 months of life in a rural district in southern Nepal. This information is important for policy makers to understand potential delays in vaccination and which populations are most at risk for targeted interventions to improve timeliness of uptake.

## 2 Methods

### 2.1 Settings and population

The setting of the study was in nine northern Village Development Committee areas in Sarlahi District, located in the central *terai* (low lying plains) region of Nepal and nested within a randomized controlled trial of maternal influenza vaccination during pregnancy [12]. At the start of the trial, prevalent pregnancies were identified through a census of all households in the catchment area. For the duration of the trial, field workers visited all households in the community where married women (15–40 years) resided every 5 weeks for surveillance of incident pregnancies. Once a pregnancy was identified women were asked for their consent to participate in the trial. Through the house-to-house surveillance, 4632 pregnancies were identified. Of these, 14 women were lost to follow-up before enrollment, 19 refused, 105 lost their fetus before enrollment, 799 were identified >34 weeks gestation (primarily at the beginning of the study), 1 had an egg allergy, and 1 intended to leave the study area and thus was not eligible. Between April 25, 2011 and September 9, 2013, 3693 pregnant women between 17 and 34 weeks gestation were randomized and vaccinated with either an influenza vaccine or placebo. All participants received ancillary benefits, which included a 90-day supply of iron-folic acid tablets, deworming medication (single dose of albendazole), clean birthing kit, chlorhexidine ointment for umbilical cord care, tetanus toxoid vaccine, if indicated, and health education messages, in addition to referral for antenatal services in the local health care system. At the time of the study, the vaccines recommended by the Nepal vaccination program in the first 6 months were BCG (at birth), OPV and DTP-Hib-HepB (both at 6, 10, and 14 weeks) (Table 1). This study was a population-based prospective cohort of infants followed from birth through 6 months post-partum. Ethical approval for the study was obtained from institutional review boards at the Johns Hopkins Bloomberg School of Public Health, the Institute of Medicine at Tribhuvan University, and Cincinnati Children's Medical Center. The trial is registered at Clinicaltrials.gov (NCT01034254).

### 2.2 Data collection

At baseline, information was collected on household structure, socioeconomic status, and demographics. At study enrollment, date of last menstrual period and pregnancy history data were collected. As soon as possible after delivery the mother and infant were visited to collect detailed birth information including infant weight and breastfeeding status. From birth through 6 months post-partum (180 days), infants were visited weekly by a field worker who recorded, based on maternal report, which specific vaccines were received in the prior 7 days. BCG is given at birth and usually results in a scar. OPV and pentavalent vaccine have the same recommended timing but differ in their administration route. The mothers reported only the type of vaccine received (not the number of the dose as this was calculated during the analysis). The field workers maintained vaccine receipt data only for the current month and therefore were not able to assess or address delays in vaccination in the field.

### 2.3 Analytic dataset

Infants were included in this analysis if they were followed for any length (0–180 days) during an approximately 3 year-period. Of 3693 women vaccinated, there were 3621 women with at least one live birth outcome. There were 3646 live born infants, 50 of whom were live-born twins and one live-born twin associated with a stillbirth. No weekly vaccination recall data were collected for 169 infants (∼5%). The final dataset consists of 3478 infants with at least one follow-up visit during the first 6 months.

Households were categorized as crowded if 5 or more people resided in the home (median number of household members). Similarly, households were dichotomized at the median into those with >2 children under 15 years versus households with 2 or fewer children under 15 years. At enrollment women reported their literacy status (binary) and pregnancy history. For parity analysis women were categorized as nulliparous or multiparous. The field workers identified the subject's ethnicity (Pahadi – a group originating from the hills or Madeshi – a group originating from north India). Twenty-five questions were asked to develop a construct to measure the socioeconomic status of households. The questions were the following: (1–3) construction materials for ground floor, first floor, and roof, (4) number of living and sleeping rooms, (5) water source, (6) type of latrine, (7) number of servants, (8–9) number of cattle and goats, (10–11) amount of *khet* and *bari* (measures of rain fed and irrigation fed arable land owned), (12–17) number of bullock carts, bicycles, motorcycles, cars/jeeps, trucks/buses, tractors, (18–23) number of clocks, radios, televisions, satellite dishes, landline phones, mobile phones, (24) electricity in home, and (25) household member working in another country. Responses for each of the 25 questions were dichotomized. The SES variable was the percent of items on the 25-item scale that were positive. If any items were missing, the score was the percent positive out of the number of non-missing items. These percentages were divided into SES quartiles for analysis. Analyses using other cut-offs of SES produced similar results to quartiles.

Gestational age was measured using a woman's report of date of last menstrual period during pregnancy surveillance (an average of 3–4 weeks recall). Gestational ages <37 complete weeks were categorized as preterm. Birthweight was collected as soon as possible after birth by study personnel using a digital scale [Tanita model BD-585, precision to nearest 10 g]. Birthweights collected >72 h after birth were excluded from the analysis of birthweight. Infants were categorized as low birthweight if weight was <2500 grams (g). Small for gestational age (SGA) was calculated using the sex-specific 10th percentile cut-off described by Alexander [13] and the INTERGROWTH-21st standards [14]. Women were asked how many hours after birth breastfeeding was initiated (if at all). Binary breastfeeding categories were created with women initiating breastfeeding within 1 h (WHO recommendation) compared to those initiating >1 h post-delivery.

### 2.4 Statistical analysis

To ensure all infants included had an opportunity to have recorded vaccinations at the recommended vaccination ages, for this analysis (excluding BCG, which is recommended at birth), infants were excluded if their lengths of follow up were less than 4 months (16 weeks) after birth (Table 1). Vaccine coverage was calculated at approximately 4 (2 week grace period after the final recommended vaccination age) and 6 months (end of study follow-up) (specifically, 112 and 180 days, respectively). The primary outcome was the proportion of infants in each vaccination dose category at 4 and 6 months.

Survival analysis was used to measure the time to vaccination for each vaccine by dose. Infants were included irrespective of length of follow-up. Kaplan–Meier curves were constructed with a specific vaccination considered the event of interest. Infants were right-censored once they had the event of interest (specific vaccine dose) or had no further follow-up recorded.

Infant, maternal, and household risk factors for time to 1st BCG, DTP-Hib-HepB (pentavalent), and OPV vaccination were analyzed using a Cox-proportional hazards model. The recommended age of first pentavalent and OPV vaccination dose, 42 days, was designated as time 0. Infants who were vaccinated prior to 42 days were assigned a date of vaccination immediately after time 0 (1 × 10^−6^). The same adjustment made for loss-to follow-up was made for those infants with no follow-up after 42 days. Infants who had at least one follow-up visit but died before 42 days were excluded from the analysis. Day of birth was used as time 0 for BCG as this vaccine is recommended at birth. For the unadjusted model, hazard ratios, 95% confidence intervals (CI), and *p*-values were reported. Risk factors measuring similar characteristics were excluded to avoid collinearity in the multivariate model. For the related variables – gestational age, birthweight, and SGA – only gestational age and SGA were included in the multivariate model. The proportionality assumption was tested through graphical diagnostics and testing based on scaled Schoenfeld residuals [15]. Time interaction terms were included for time-varying coefficients that were statistically significantly associated with time to vaccination in the bivariate model. The multivariable model included adjusted hazard ratios, 95% CIs and *p*-values.

Statistical significance was set at *p* < 0.05 for all testing. All statistical analyses were conducted in Stata/SE 14.0 (STATA Corp., College Station, TX).

## 3 Results

In this study, 3478 infants were visited at least once from birth through 180 days of life. The visit dates ranged from May 24, 2011 to April 29, 2014. The mean age at last follow-up visit was 167 days (range: 3–180 days). The average length of follow-up was 156 days. For DTP-Hib-HepB (pentavalent) and OPV vaccination coverage estimates, 168 infants were excluded due to having no data at or beyond 4 months (2 weeks past the recommended age of 3rd infant vaccine dose). Common reasons for no further follow-up past age four months included death, a temporary move of the mother and her newborn to her mother's house, or a permanent move from the study area. Altogether, 3310 infants were observed to at least age 4 months. Of these included infants, 23% (*n* = 752) had received no vaccinations of any type by age six months.

**Fig. 1:**
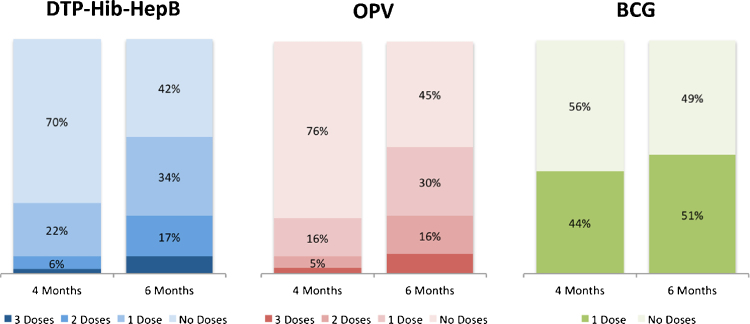
Infant immunization coverage at 4 and 6 months. *Excludes infants not observed past 4 months.

The majority (70%) of infants had received no pentavalent immunization by age 4 months, two weeks past the recommended age for completion of all 3 doses (Fig. 1). By age 6 months, 42% of infants had received no pentavalent vaccinations, with only 7% fully vaccinated by 6 months. An even higher percentage (76%) of infants had no OPV vaccination by 4 months with a minority (8%) fully vaccinated by age 6 months. By age 6 months only half (49%) of children had received a BCG vaccine, which is recommended at birth. A minority of infants were vaccinated prior to the recommended vaccination ages. For pentavalent vaccine 3%, 0.2%, and 0% received the first, second, and third doses early, respectively. For OPV early vaccination receipt was found for 5%, 0.2%, and 0.03% for the first, second, and third doses, respectively.

**Fig. 2:**
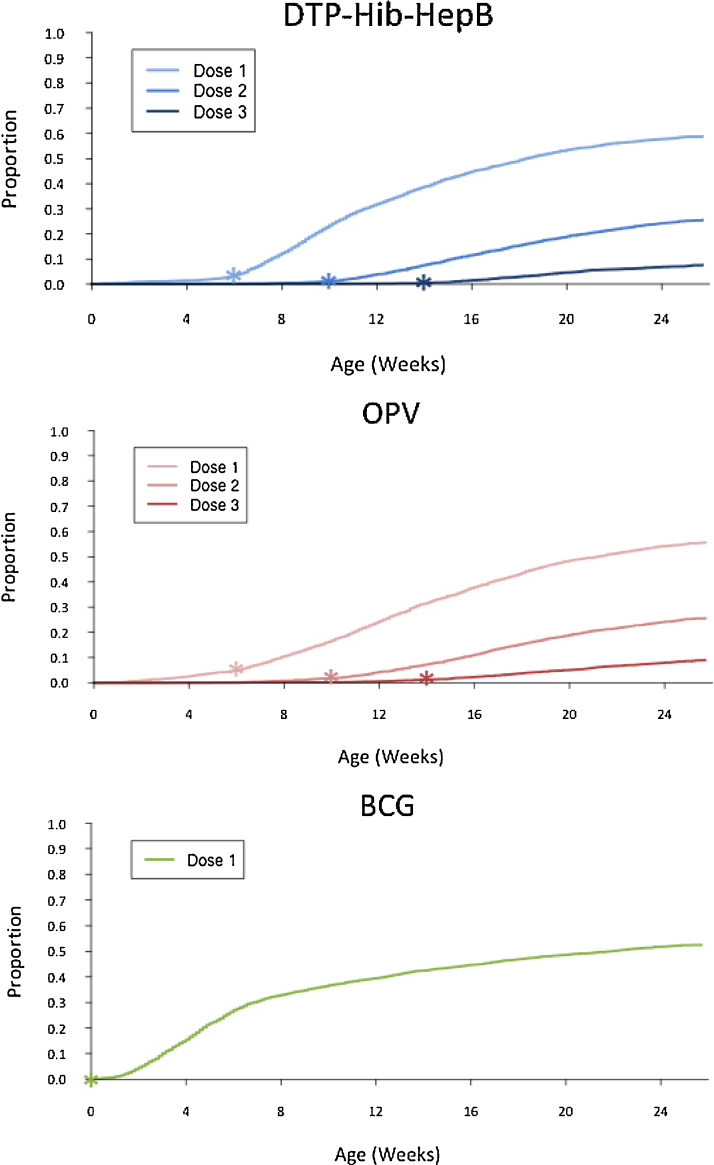
Time to immunization. *Stars indicate the recommended age for each vaccine dose.

The median age at first DTP-Hib-HepB vaccination, estimated using survival curves, was 18.3 weeks (95% CI: 17.6–19.1) (Fig. 2). The median age of first OPV was 21.1 weeks (95% CI: 20.0–22.1); BCG median was 21.9 weeks (95% CI: 19.6–23.7).

Cox proportional hazard models were used to estimate the relative hazard of being unvaccinated in unadjusted (bivariable) and adjusted (multivariable) models (Table 2, Table 3, Table 4). Highly collinear or related variables were excluded from the adjusted models.

For time to first pentavalent dose, the strongest associations in the bivariable models were for ethnicity (HR 1.34; 95% CI: 1.22–1.48), delayed breastfeeding initiation (HR 1.19; 95% CI: 1.08–1.31) and number of children under 15 years for infants older than 10 weeks (HR 1.43; 95% CI: 1.14–1.80) (Table 2). In the multivariable model ethnicity, breastfeeding, and number of children under 15 years for the period when infants were >10 weeks of age remained statistically significant.

For time to first OPV dose, the strongest associations for highest hazard of being unvaccinated in bivariable models were for high number of children <15 years (HR 1.21; 95% CI: 1.09–1.34) and maternal illiteracy (Table 3). Maternal illiteracy increased the hazard of being unvaccinated by 3% per week of infant age (95% CI: 1–6%). In the multivariable model these two factors remained statistically significantly associated with OPV vaccination delay.

For time to first BCG dose, the strongest factors associated with a higher hazard of being unvaccinated in bivariable models were for ethnicity (HR 1.26; 95% CI: 1.15–1.39) and illiteracy (HR 1.16; 95% CI: 1.05–1.29) (Table 4). Ethnicity was the only factor statistically significantly associated with BCG vaccination delay in the multivariate model. We examined whether delivery location (hospital/clinic versus home birth) was associated with delay (data not shown) given BCG is recommended at birth; there was no association between delivery location and BCG timing.

## 4 Discussion

The current approach to measuring coverage of immunization programs by assessing the proportion of children immunized among those 12–23 months of age does not address the important question of when the scheduled vaccines were administered. In this prospective, community-based study, delays in immunizations in infants <6 months of age in Sarlahi District, Nepal, were common, at a time when infants are at highest vulnerability for morbidity from these infections. These significant delays were not captured by WHO estimates of vaccination coverage. Nepal data from 2012 show DTP1 and DTP-Hib-HepB3, and OPV3 coverage all at 90%, and BCG coverage at 96% among children age 12–23 months [16]. The 2011 Nepal Demographic and Health Survey (DHS) estimates vary slightly from this with DTP1 and DTP3 coverage at 96% and 91%, respectively [17]. OPV1 coverage was 97% versus 92% for OPV3. BCG coverage was 97%. From the same 2011 survey, in the central *terai* region, where Sarlahi District is located, 96%, 92%, and 87% of children were reported to have received one, two, and three doses, respectively, of DTP by ages 12–23 months [17]. Coverage for OPV1-3 was 96%, 92%, and 89%, respectively. BCG coverage was 96%. While these coverage estimates are high, our data show many infants receive vaccines on a delayed schedule and thus are at increased risk for vaccine-preventable diseases. Globally, WHO and UNICEF use officially reported data and sample survey data to measure vaccination coverage of children 12–23 months [3]. As a result, if there are significant delays in vaccination, but vaccines are complete by age 2, a child is still considered as vaccinated on schedule. Similarly, in the U.S. and elsewhere, standard national reporting statistics obscure delays during periods when infants are most at risk for vaccine-preventable diseases [11], [18], [19], [20]. National-level reporting may also mask within-country variation in vaccination timeliness [11].

Our finding of significant vaccination delay is consistent with data from other countries. For example, Japan's reported DTP3 coverage was 98% in 2013, however data from a representative city in Japan showed less than 50% DTP coverage by age 12 months [19]. In the U.S., a study found almost half of children had some delay in receiving a DTaP vaccine dose and 16% were delayed in vaccine receipt for more than 6 months in the first two years; 32% had some delay in receipt of poliovirus vaccine, with 9% at least 6 months delayed [18] despite national DTP3 and poliovirus vaccine coverage over 90% in 2013 [21]. A longitudinal study in Ghana reported that while DTP3 coverage was 95% at 12 months, only 10% of infants were vaccinated within 1 week of the scheduled time (14 weeks); the median delay for DTP3 was 4 weeks [22]. In the same study BCG coverage was 98% at 1 year but only 38% of infants were vaccinated within the first week of life; the median delay for BCG was 1.7 weeks; similar delays were found for one coastal Kenyan district [23]. In an Indian study using vaccination card records only 31% of infants received DTP3 by 14 weeks [24]. A study examining the timing of vaccination in low and middle income countries, based on surveys and imputed data, found at 6 months median coverage was 82% (Interquartile Range [IQR]: 67–89%) for DTP1, 36% (IQR: 23–54) for DTP3, and 85% (IQR: 73–91) for BCG [11]. Our data from Sarlahi, Nepal of DTP1 (57%), DTP3 (7%), and BCG (49%) coverage at 6 months are lower than these estimates from other similar countries. An interpretation of this is that the rural *terai* of Nepal may have an increased vaccination delay compared to other parts of the country or similar countries. However, Sarlahi's vaccination coverage data, measured later at 12–23 months, are comparable to that of Nepal as a whole. Survey and imputed data in general may lead to an overestimation of coverage. While the weekly vaccination recall could have prompted parents to immunize thus biasing our results in the direction of better timeliness, our prospective weekly active surveillance data are potentially a more precise and unbiased estimation of timing of vaccination coverage than previous estimates.

Despite the vaccination delays found in our study, the National Immunization Program is a high priority program in Nepal, with the country already having achieved Millennium Development Goal 4 on child mortality reduction [25], [26]. Immunization delay is important as it leaves infants at risk for vaccine preventable diseases potentially contributing to morbidity and mortality [7], [27]. For example, children who are unimmunized or under immunized are at increased risk for pertussis and pertussis hospitalization compared to their more fully immunized peers [19], [28], [29], [30], [31], [32].

In our multivariate models, Madeshi ethnicity was associated with an approximately 20% increased hazard of delay for DTP-Hib-HepB1 and BCG vaccines. A high number of children in the household was a risk factor for delay in the first pentavalent and OPV vaccines with the effect of crowding increasing with increasing age for pentavalent vaccine. Factors associated with only one vaccine combination were breastfeeding initiation for DTP-Hib-HepB1 and literacy for OPV1 with literacy's association with OPV1 delay increasing with infant age. One reason why these factors might contribute to vaccination delay is that they are markers for decreased access or utilization of health services. Mothers who have lower utilization of antenatal care might have had less exposure to the importance of early initiation of breastfeeding. Women of Madeshi ethnicity have less mobility and empowerment, and are therefore less likely to access health care resources for themselves and their children. The demands of more than 2 children <15 years in a household might limit the time and resources available for well child visits. Together, these factors might lower access to visits where infants have an opportunity for vaccination. We found no difference in vaccination status by sex, birth order (parity), and SES, of which the latter two are in contrast to that found in the 2011 Nepal DHS [17]. The lack of observed sex differences is generally consistent with previous global studies [33] although Nepal's neighbor, India, has observed sex differences in vaccination coverage [34]. Surprisingly, for BCG we saw no association between facility versus home delivery location in vaccination timing demonstrating a failure in the health system to vaccine infants while still in the delivery facility.

Reasons for vaccination delay in low and middle-income countries include poor immunization supply, lack of access to health services, and family characteristics [20], [22], [35]. Parents may also be hesitant to vaccinate or not view the costs involved with vaccination worth the benefit. Infants in Ghana who were poorer, had less educated mothers, and lived in rural versus urban areas were significantly more likely to delay vaccination compared to urban infants whose mothers were educated and in a higher income groups [22]. A study of 31 low and middle income countries also found that children in poorer families and families with more than one child were at increased risk for vaccination delay [20]. In the U.S. vaccination delay is associated with a mother who is unmarried, less educated, non-Hispanic black, and uses public vaccination providers [18]. In contrast to these studies, in our Nepal population, low socioeconomic status was not a significant predictor in vaccination delay. Our findings are similar to previous findings with maternal literacy and number of children in the household both being significant predictors of vaccination delay. Our study provides an improved understanding of Nepal-specific factors contributing to vaccination delay that can help programs focus on at-risk populations to increase on-time vaccination.

A limitation of our study is that our surveillance extended only for the first 6 months of life. We were not able to capture the timing of vaccination receipt after age 6 months to age 12 months. We cannot provide if or when vaccines were received to capture the full delay. This limited the direct comparability of our data to official data reported at age 1 year. The most likely explanation is that there is catch-up of vaccination beyond 6 months of age. Official reporting in some countries may overestimate the coverage in part to reach donor targets such as GAVI's immunization services support (ISS) [3]. However, that study found that Nepal did not over report its performance during the period when GAVI incentives were provided.

Another limitation of our study was that recording of vaccine receipt was reported by parents and not confirmed by review of immunization cards. This could have led to misclassification if the parent reported an incorrect vaccine. However, the two vaccines with the same schedule (OPV and pentavalent) differ in their administration (oral and injection) so this misreporting is unlikely. It is possible BCG and pentavalent vaccines could have been confused, but BCG is given soon after birth, usually produces a typical scar not seen with other vaccines, and is done at a different injection site than pentavalent vaccine. Overestimation of coverage could have occurred if parents over reported vaccine receipt or underestimation if parents forgot or were unaware of a vaccine the infant previously received. However, parents were visited in their homes on a weekly basis limiting the chance for recall bias.

Strengths of this study include the population-based cohort study design that followed infants prospectively on a weekly basis from birth through age 6 months. The capture of time of vaccination provides important information for Nepal policy makers. While the population was limited to one area of Sarlahi district in Nepal, the results are likely generalizable to much of the rural Nepalese population. The majority of the Nepali population lives in the *terai* region, where Sarlahi District is located and infant health and vaccination indictors are similar to country-wide estimates [17].

## 5 Conclusion

We found significant delays in receipt of recommended infant vaccinations in a prospective population-based cohort in southern Nepal. The standard approach to immunization coverage estimates worldwide does not fully capture the excess vaccine-preventable disease risk attributable to delays in vaccination. Timeliness of routine childhood immunization should be emphasized to reduce infant morbidity and mortality risk from vaccine-preventable diseases. Age appropriate vaccination indicators should be considered as another metric of an immunization program's impact.

## Figures and Tables

**Table 1:** Nepal immunization schedule during study period May 2011–April 2014.

Vaccine	Age of administration
BCG	At birth
DTP-Hib-HepB	6 weeks, 10 weeks, 14 weeks
OPV	6 weeks, 10 weeks, 14 weeks
MR	9 months
JE	12–23 months (high risk districts)
TT	During pregnancy
Vitamin A	6–59 months

**Table 2:** Risk factors for delay in time to first DTP-HepB-Hib vaccination.

Risk factor	No.	%	Cox proportional hazard model					
			Unadjusted			Adjusted		
			HR^a^	95% CI^b^	*p*-Value^c^	HR	95% CI	*p*-Value
Sex								
Female	1645	47%						
Male	1829	53%	1.07	0.98–1.18	0.13	1.03	0.93–1.15	0.57
Gestational age^d^								
Term	3045	88%						
Preterm	428	12%	1.11	0.96–1.28	0.16	1.17	0.98–1.40	0.08
Birthweight^e^								
Normal	2036	75%						
Low birthweight	668	25%	1.14	1.01–1.28	0.04			
Small-for-gestational age^f^								
Non-SGA (IG)	1587	63%						
SGA (IG)	939	37%	1.09	0.98–1.22	0.11			
Non-SGA (A)	1427	52%						
SGA (A)	1296	48%	1.11	1.00–1.23	0.05	1.09	0.97–1.22	0.13
Breastfeeding								
Breastfed <1 h	1192	35%						
Non-breastfed 1st hour	2168	65%	1.19	1.08–1.31	<0.01	1.16	1.04–1.29	0.01
Literacy								
Literate	1967	61%						
Illiterate	1250	39%	1.12	1.01–1.23	0.03	1.05	0.92–1.18	0.48
Parity								
Non-first pregnancy	2021	58%						
First pregnancy	1446	42%	1.10	1.00–1.20	0.05	1.09	0.97–1.23	0.13
Ethnicity								
Pahadi	1929	58%						
Madeshi	1412	42%	1.34	1.22–1.48	<0.01	1.21	1.07–1.37	<0.01
SES^g^								
Lower vs. higher	3342		1.02^i^	0.98–1.07	0.30			
Crowding^h^								
Uncrowded	1817	55%						
Crowded	1492	45%	0.98	0.89–1.07	0.64			
Children under 15 years								
Age 6–10 weeks								
≤2 children	1895	57%						
>2 children	1414	43%	1.05	0.94–1.17	0.38	1.02	0.90–1.16	0.72
Age >10–26 weeks								
≤2 children	1895	57%						
>2 children	1414	43%	1.43	1.14–1.80	<0.01	1.45	1.11–1.90	<0.01

a Hazard ratio; interpretation: ratio of the hazard of being unvaccinated in risk group compared to the reference group.

b 95% confidence interval.

c *p*-Values calculated from the Wald test of the maximum likelihood estimate (MLE) of the coefficient.

d Gestational age: preterm (<37 weeks), term (≥37 weeks).

e Birthweight: low birthweight (<2500 g), normal (≥2500 g).

f Small-for-gestational age: IG = INTERGROWTH-21st standards; A = Alexander standards.

g Socioeconomic status (SES): average of 24 SES measures categorized into quartiles and modeled as a continuous variable (1–4).

h Crowding: crowded (≥5 persons living in household), uncrowded (<5 persons living in household).

i Interpretation example: hazard of being unvaccinated in the bottom quartile compared to 2nd lowest quartile.

**Table 3:** Risk factors for delay in time to first OPV vaccination.

Risk factor	No.	%	Cox proportional hazard model					
			Unadjusted			Adjusted		
			HR^a^	95% CI^b^	*p*-Value^c^	HR	95% CI	*p*-Value
Sex								
Female	1645	47%						
Male	1829	53%	1.03	0.93–1.13	0.55	1.03	0.92–1.15	0.62
Gestational age^d^								
Term	3045	88%						
Preterm	428	12%	1.16	0.99–1.35	0.06	1.14	0.94–1.37	0.18
Birthweight^e^								
Normal	2036	75%						
Low birthweight	668	25%	1.07	0.94–1.22	0.30			
Small-for-gestational age^f^								
Non-SGA (IG)	1587	63%						
SGA (IG)	939	37%	1.04	0.92–1.17	0.53			
Non-SGA (A)	1427	52%						
SGA (A)	1296	48%	1.09	0.97–1.21	0.14	1.10	0.98–1.24	0.12
Breastfeeding								
Breastfed <1 h	1192	35%						
Non-breastfed 1st hour	2168	65%	1.14	1.03–1.26	0.01	1.05	0.94–1.18	0.37
Literacy								
Age 6 weeks								
Literate	1967	61%						
Illiterate	1250	39%	0.96	0.78–1.17	0.66	0.89	0.70–1.13	0.34
1 week increase in age								
Literate	1967	61%						
Illiterate	1250	39%	1.03	1.01–1.06	<0.01	1.03	1.00–1.06	0.03
Parity								
Non-first pregnancy	2021	58%						
First pregnancy	1446	42%	0.96	0.87–1.06	0.43	0.99	0.88–1.12	0.93
Ethnicity								
Age 6 weeks								
Pahadi	1929	58%						
Madeshi	1412	42%	1.17	0.96–1.43	0.13	1.20	0.94–1.54	0.15
1 week increase in age								
Pahadi	1929	58%						
Madeshi	1412	42%	1.03	1.00–1.05	0.02	1.01	0.98–1.04	0.51
SES^g^								
Lower vs. higher	3342		1.02^i^	0.98–1.07	0.39			
Crowding^h^								
Uncrowded	1817	55%						
Crowded	1492	45%	1.00	0.90–1.10	0.95			
Children under 15 years								
≤2 children	1895	57%						
>2 children	1414	43%	1.21	1.09–1.34	<0.01	1.13	1.00–1.28	0.05

a Hazard ratio; interpretation: ratio of the hazard of being unvaccinated in risk group compared to the reference group.

b 95% confidence interval.

c *p*-Values calculated from the Wald test of the maximum likelihood estimate (MLE) of the coefficient.

d Gestational age: preterm (<37 weeks), term (≥37 weeks).

e Birthweight: low birthweight (<2500 g), normal (≥2500 g).

f Small-for-gestational age: IG = INTERGROWTH-21st standards; A = Alexander standards.

g Socioeconomic status (SES): average of 24 SES measures categorized into quartiles and modeled as a continuous variable (1–4).

h Crowding: crowded (≥5 persons living in household), uncrowded (<5 persons living in household).

i Interpretation example: hazard of being unvaccinated in the bottom quartile compared to 2nd lowest quartile.

**Table 4:** Risk factors for delay in time to BCG vaccination.

Risk factor	No.	%	Cox proportional hazard model					
			Unadjusted			Adjusted		
			HR^a^	95% CI^b^	*p*-Value^c^	HR	95% CI	*p*-Value
Sex								
Female	1645	47%						
Male	1829	53%	0.96	0.88–1.06	0.45	0.92	0.83–1.03	0.13
Gestational age^d^								
Term	3045	88%						
Preterm	428	12%	1.12	0.97–1.30	0.12	1.08	0.91–1.28	0.38
Birthweight^e^								
Normal	2036	75%						
Low birthweight	668	25%	1.03	0.92–1.16	0.62			
Small-for-gestational age^f^								
Non-SGA (IG)	1587	63%						
SGA (IG)	939	37%	1.04	0.93–1.16	0.51			
Non-SGA (A)	1427	52%						
SGA (A)	1296	48%	1.02	0.92–1.13	0.66	1.03	0.93–1.15	0.56
Breastfeeding								
Breastfed <1 h	1192	35%						
Non-breastfed 1st hour	2168	65%	1.14	1.04–1.26	0.01	1.05	0.94–1.18	0.36
Literacy								
Literate	1967	61%						
Illiterate	1250	39%	1.16	1.05–1.29	<0.01	1.08	0.96–1.22	0.22
Parity								
Non-first pregnancy	2021	58%						
First pregnancy	1446	42%	1.01	0.92–1.11	0.81	0.96	0.86–1.08	0.52
Ethnicity								
Pahadi	1929	58%						
Madeshi	1412	42%	1.26	1.15–1.39	<0.01	1.17	1.04–1.33	0.01
SES^g^								
Lower vs. higher	3342		1.01	0.97–1.05	0.74			
Crowding^h^								
Uncrowded	1783	54%						
Crowded	1529	46%	1.02^i^	0.93–1.13	0.63			
Children under 15 years								
≤2 children	1897	57%						
>2 children	1415	43%	1.02	0.93–1.12	0.68	0.95	0.85–1.06	0.36

a Hazard ratio; interpretation: ratio of the hazard of being unvaccinated in risk group compared to the reference group.

b 95% confidence interval.

c *p*-Values calculated from the Wald test of the maximum likelihood estimate (MLE) of the coefficient.

d Gestational age: preterm (<37 weeks), term (≥37 weeks).

e Birthweight: low birthweight (<2500 g), normal (≥2500 g).

f Small-for-gestational age: IG = INTERGROWTH-21st standards; A = Alexander standards.

g Socioeconomic status (SES): average of 24 SES measures categorized into quartiles and modeled as a continuous variable (1–4).

h Crowding: crowded (≥5 persons living in household), uncrowded (<5 persons living in household).

i Interpretation example: hazard of being unvaccinated in the bottom quartile compared to 2nd lowest quartile.
